# Exploring Ecosystems and Health by Shifting to a Regional Focus: Perspectives from the Oceania EcoHealth Chapter 

**DOI:** 10.3390/ijerph121012706

**Published:** 2015-10-13

**Authors:** Jonathan Kingsley, Rebecca Patrick, Pierre Horwitz, Margot Parkes, Aaron Jenkins, Charles Massy, Claire Henderson-Wilson, Kerry Arabena

**Affiliations:** 1 Melbourne School of Population and Global Health, The University of Melbourne, Parkville 3010, Australia; 2 School of Health & Social Development, Deakin University, Melbourne 3125, Australia; E-Mail: rebecca.patrick@deakin.edu.au; 3 School of Natural Sciences, Edith Cowan University, Joondalup 6027, Australia; E-Mail: p.horwitz@ecu.edu.au; 4 School of Health Sciences, University of Northern British Columbia, Prince George V2N4Z9, Canada; E-Mail: Margot.Parkes@unbc.ca; 5 School of Natural Sciences, Edith Cowan University, Joondalup 6027, Australia; E-Mail: a.jenkins@ecu.edu.au; 6 Fenner School for Environment and Society, Australian National University, Canberra 2601, Australia; E-Mail: charles.massy@anu.edu.au; 7 School of Health & Social Development, Deakin University, Melbourne 3125, Australia; E-Mail: claire.henderson-wilson@deakin.edu.au; 8 Melbourne School of Population and Global Health, The University of Melbourne, Parkville 3010, Australia; E-Mail: kerry.arabena@unimelb.edu.au

**Keywords:** Oceania region, ecosystems approaches to health

## Abstract

This article highlights contributions that can be made to the public health field by incorporating “ecosystem approaches to health” to tackle future environmental and health challenges at a regional level. This qualitative research reviews attitudes and understandings of the relationship between public health and the environment and the priorities, aspirations and challenges of a newly established group (the Oceania EcoHealth Chapter) who are attempting to promote these principles. Ten semi-structured interviews with Oceania EcoHealth Chapter members highlighted the important role such groups can play in informing organisations working in the Oceania region to improve both public health and environmental outcomes simultaneously. Participants of this study emphasise the need to elevate Indigenous knowledge in Oceania and the role regional groups play in this regard. They also emphasis that regional advocacy and ecosystem approaches to health could bypass silos in knowledge and disciplinary divides, with groups like the Oceania EcoHealth Chapter acting as a mechanism for knowledge exchange, engagement, and action at a regional level with its ability to bridge the gap between environmental stewardship and public health.

## 1. Background

“The bottom line is that we need to conceive of ourselves as an integral part of the eco-community on this planet or we are to perish” [1]. Eco-community refers to individuals who understand the intricate relationship between all Earth-human-animal-metaphysical elements and recognise the diversity of such a complex union [2].

Humans seem to be ignoring the evidence that the loss of species and ecosystems represents an unparalleled set of global public health challenges [3]. Academics and practitioners believe that such issues can be managed by integrating themselves into ecological systems, from wetland ecosystems [4] to urban sprawls [5], because it will directly and indirectly impact on health [6]. Parkes [7] argues that “ecosystems tend to receive little attention, despite providing a non-negotiable basis for the food, water, livelihoods and living systems on which we depend”. Arabena and Kingsley [8] highlight that part of the problem is that humans continue to employ monoculture actions that degrade ecosystems.

Literature recommends that health practitioners accept that both human and non-human systems are central to well-being going beyond “traditional health approaches” that promote the relationship humans have to their ecosystems [9]. Horwitz and Finlayson [4] explain this by “describing the condition of an entire… ecosystem… [It] might be applied in a series of tiers: the health of an individual, the health of a population, the health of an ecosystem, each nested within the next tier.” Many Indigenous cultures have organized society and culture along these principles, including Aboriginal and Torres Strait islanders, who understand the word ‘health’ as the compatibility between life and land [7,10,11].

This paper attempts to gain a better understanding of such approaches by capturing the priorities, aspirations and challenges of an emerging group (the Oceania EcoHealth Chapter) that are attempting to incorporate ecosystem approaches to health. Specifically, to explore the relationship between public health and the environment and implications for the Oceania EcoHealth Chapter. The research questions this article address: what are the benefits of focusing on ecosystem approaches to health at a regional level? What is already being done? What challenges does this approach bring and what institutional structures are required to incorporate such methods in Oceania?

The Oceania EcoHealth Chapter aims to consult, represent and engage cultural perspectives of its membership and advocate local/regional issues (MOU between the International Association for Ecology and Health (IAEH; also EcoHealth) and Oceania EcoHealth Chapter, 2013). The Oceania EcoHealth Chapter is made up of Indigenous and non-Indigenous academics attempting to progress ecosystem approaches to health regionally. The Chapter aligns with international organisations like the IAEH whose mandate is to “strive for sustainable health of people, wildlife and ecosystems” [12].

Ecosystem approaches were first applied in academic writing in 1978 and in public health literature in the 1990’s [13] but there is evidence of integration earlier. Within the Western scientific tradition, the Greek physician Hippocrates published the notion of human health being contextually dependent on environment around 2600 years ago. Recently in public health (with an emphasis on health promotion) this has been represented in Bronfenbrenner’s *Ecological Framework for Human Development* [14], Hancock and Perkins *Mandala of Health* [15], and the *Ottawa Charter* [16] that identified social-ecological approaches. Ecosystem approaches to health have been strengthened through the emerging field of EcoHealth [13].

EcoHealth involves research and practice to promote sustainability of individuals, animals and biodiversity by linking complex interaction of ecosystem, socio-cultural and economic factors [17,18,19]. Ecosystem approaches to health identify *sustainability* as the central focus of global health *rather* than sustainable development, which is a paradigm of economic imperative [19]. EcoHealth “place[s] disease dynamics in the broader contexts of sustainable agriculture, socio-economic development, environmental protection… and the complex patterns of global change” [9].

Transdisciplinarity is fundamental to EcoHealth, allowing for multiple perspectives and collaboration between stakeholders to tackle ‘complex societal challenges’ [20,21]. This ensures EcoHealth crosses disciplinary divides by promoting ‘collective endeavour as a “transdisciplinary imperative for a sustainable future’’’, providing a mechanism for respect, integration and engagement [22]. Max-Neef [23] differentiated between disciplinarity, multidisciplinarity, interdisciplinarity, and transdisciplarity, explaining the deeper notion of being transdisciplinary is achieving a “quantum logic” beyond our current “linear logic” and assumptions of a “single reality”. Max-Neef [23] identified that this requires different perspectives and approaches that are more systemic and holistic than are currently applied in Western science. Parkes *et al.* [22] explains that EcoHealth research goals include sustainability, equity, and the integration of scientific knowledge that translates across the research, policy and community sector attempting to apply strong transdiciplinarity [23]. Lovell *et al.* [24] identified that disciplines that support ecosystem approaches to health should build stronger advocacy platforms at local and global levels with scholars recently emphasising the target audience of EcoHealth being the general public [20].

One defining feature of ecosystem approaches to health is the incorporation of Indigenous diverse knowledge systems. Saint-Charles *et al.* [20] and Parkes [7] notes EcoHealth aligns with Indigenous worldviews due to the recognition of the complex interconnection that ecosystems play in human health. For example, the Aboriginal Australian term “Ngurra-Kurlu” is a holistic framework for environmental management, cross-cultural learning, fostering ecosystem stewardship to improve health, and well-being [25]. Throughout Oceania, this holistic and ecological knowledge is linked to improved environment– and health–related outcomes with research indicating engagement with Indigenous communities critical to policy development and decision-making [26,27,28]. Such holistic approaches require local and regional understandings of ecosystem approaches, the premise for the development of the Oceania EcoHealth Chapter, because:

“The development of ecohealth approaches that address health more holistically, and encourage integration and exchange among multiple forms of knowledge, suggest a new terrain of research and practice that can greatly benefit from, and potentially be highly complementary to, holistic approaches to Aboriginal and Indigenous health” [7].

## 2. Regionalism and Oceania

Regions encompass diverse social relationships and geographical location, however, literature emphasises regional governance as if “closed, bounded and coherent with shared… interests” [29]. Some believe the success of regionalism is based on clear mandate, partnerships, leadership, and vision extending to the public [30]. Nonetheless, regional environment initiatives have typically been short-term rather than applying ‘social-ecological processes’ and long-term community engagement strategies [31,32]. Across Oceania, regional plans have been developed to tackle ecological issues with limited success, in part due to lack of incorporation of diverse local voices [27,28,33]. Regionalisation in health literature has been described as reducing costs, increase efficiency, citizen participation, and accountability, though it is also associated with instability, and lacking long-term approaches and community involvement [30].

Oceania is made up of small islands (excepting Australia, New Zealand, and Papua New Guinea (PNG)) with the ocean a fundamental feature of this diverse region [26,27,34,35,36,37,38]. Except these larger countries, Oceania encompasses islands in Melanesia, Micronesia and Polynesia that cover 1000 inhabited islands of nearly 30,000 [26,27,31,39]. Excluding PNG, Fiji, Australia, and New Zealand, the United Nations classify these islands as Small Island States [31,40]. These islands’ unique ecosystems play an integral economic, social, and health role and include habitat descriptions such as volcanic, fertile, tropical, rainforests, limestone, atolls, woodlands, savannahs, freshwater lakes/streams, salt marshes, mudflats, mangroves, and offshore coral reefs [26,27,31,37]. The inherent characteristic of the region, and hence the name, is that it is intimately connected with ocean processes.

Historically, Indigenous people arrived in PNG at 60,000 B.C.E. [27], in Australia 70,000 years ago [41], and isolated islands as late as 1000 C.E. [27,31]. Diverse Indigenous cultures continue to sustain traditions and ecological adaptations that have shaped the Oceania region [27,42]. However, there are now extra pressures on populations living in the Oceania with stress associated with, for example, ecosystem resources, food security, non-communicable diseases and poverty [37,38,43].

Oceania provides a seedbed of learning for EcoHealth because of its “extraordinary” ecological, cultural, and social diversity coupled with among the highest incidence of environmental disasters [26,28,39,44]. Climate change is predicted, for example, to increase vector-borne (in Australia, New Zealand, Cook Islands, Fiji, Tongue, Solomon Islands, Marshall Islands, Palau), diarrhoeal (in Australia, Cook Islands, Fiji, Tonga, Tuvalu, Niue), and water-borne diseases (in Federation State of Micronesia, Kirbati, Marshall Islands, Vanuatu) [39,43]. These issues are associated with rising sea levels, decreased freshwater, marine and coastal health and increased likelihood of droughts, floods, earthquakes, cyclones, and ocean acidification [26,28,31,37,44,45,46]. Such vulnerability is expected to lead to an influx of environmental refugees to Australia and New Zealand and though both have built political ties within Oceania [39], this may lead to social and political tensions. These developed economies are often perceived to negatively impact poorer countries that will be disproportionally affected by ecological and health concerns [31,33,38,39,44,47].

Previous strategies in Oceania have lacked the capacity to tackle ecological issues with success “limited in scope, with a few patchy conservation outcomes, despite the dedication of many” [26]. Further, regional policies are not linking ecosystems with health [27], although specific recommendations to do so have been made in recent literature with regard to Integrated Island Management and in respect to wetland management surrounding natural disasters [36,48]. Literature recommends testing regional, local or global approaches, although regional approaches in Oceania have been criticized [28,32,33]. Granthem *et al.* [28] emphasised the importance of governance structures in Oceania applying small-scale, bottom-up approaches that engage local communities, concluded:

“The effectiveness of top-down versus bottom up planning will depend on how well it is matched with governance infrastructure and resources… Within tropical Oceania, there may be scope to scale up local level management… similarly, there may be scope to scale down top-down regional plans where appropriate… Transformative policies addressing drivers of environmental degradation… will be required if countries are to adequately respond to and reduce non-climatic threats to ecosystems”

The current intergovernmental body able to make policy changes is the Pacific Islands Forum Secretariat [33,44], where more practical change can be driven by South Pacific Regional Environment Program and the Secretariat of the Pacific Community, assisted by local civil society organizations. There is debate over the success of the Forum due to scepticism of its leadership and governance capability [33]. Maclellan [33] noted that Australian and New Zealand input make it hard for “regional consensus” with groups now considering alternative models to “mobilize” Oceania through integrating Indigenous and Western modes of science, and replacing the notion of Small Island States with “Large Ocean Countries” that emphasises the importance of oceans and increasing locally-driven responses.

## 3. Case Study: The Oceania EcoHealth Chapter

Public health is defined as “the science and art of promoting health, preventing disease, and prolonging life through organized efforts of society” [49]. Due to events of the nineteenth century through isolation of diseases, discovery of anaesthesia, and improvements in sanitation, public health is often associated with biomedical models [50,51]. The authors of this paper believe a new vision is required, emphasising cooperative and democratic action at all levels of society based on the principle of planetism and well-being for every person on this Earth, a principle that asserts that we must conserve, sustain, and make resilient the planetary and human systems on which health depends by giving priority to the wellbeing of all [52].

Forming regional representative bodies supports Lovell and colleagues’ [24] recommendation that effective research, policy, and practice requires geographically-appropriate and landscape-specific actions to tackle global environmental challenges. Further, Arabena [1] urged EcoHealth practitioners to take control of government policy formulation and implementation, developing frameworks to tackle ecological, economic, and health agendas by moving away from silos to more holistic approaches. An example of this in action is in Canada with EcoHealth networks developed to create community hubs, develop research capacity, influence policy and practice, to ensure learning beyond isolated projects, and to foster innovation [21].

In 2012, The Oceania EcoHealth Chapter was formed at the 4th Biennial IAEH Conference in Kunming (China) as members felt they needed local responses and regional engagement to ecosystem approaches to health. This lack of representation was evident in pre-workshops held at the conference where diverse regions, like Europe and Oceania, were merged to discuss local EcoHealth priorities [20].

The Chapter, although in the formative stage, gained momentum when delegates attended the inaugural Oceania EcoHealth Symposium (December, 2013). The symposium brought leading academics, farmers, Indigenous and like-minded individuals, together to sign the inaugural MOU between the IAEH and Oceania EcoHealth Chapter; apply collective learning to explore how the group could develop; and promote diverse knowledge systems. Data collection was undertaken at this event as it provided a setting for Oceania EcoHealth Chapter members to explore the relationship between public health and the environment and perspectives on regional approaches.

## 4. Method

Qualitative methodology was applied in this research to better understand the priorities, aspirations and challenges of the Oceania EcoHealth Chapter, ecosystem approaches to health as applied in the public health field and opportunities of such approaches in Oceania. Data was collected during and two weeks following the Oceania EcoHealth Symposium through semi-structured interviews with Oceania EcoHealth Chapter members. This method required an interviewer asking a series of questions through an interview guide to illicit open discussions around a topic [53].

The authors of this paper were all contributing members of the Oceania EcoHealth Chapter attempting to strengthen this group, reduce the gap in the research, and develop better solutions in the region [53]. This can be viewed as both a strength and limitation of this research due to the immersed nature of the authors, which aligns with the ethnographic approach applied by the lead researcher [53,54]. Prior to collecting and analysing data, approval was received from the Deakin Human Research Ethics committee (project number: H96_2013) to undertake this research.

Purposive sampling was applied to select participants, who identified as Oceania EcoHealth Chapter members, from a range of stages of careers and cultural backgrounds. Purposive sampling involves selecting participants who are “able to provide the desired information” of a group [55]. 10 semi-structured interviews were undertaken to provide a snapshot of attendees (n = 100) at the Oceania EcoHealth Symposium. Table 1 highlights participants’ demographics—the authors recognise this sample is not reflective of the region but more the Oceania EcoHealth Chapter.

**Table 1:** Participants’ demographics.

Gender	5 Males; 5 Females
Profession	6 academics; 1 medical practitioner; 1 self-employed nature therapist; 1 farmer; 1 conservationist
Geographical location	6 Australia (2 NSW/Victoria, 1 ACT/WA); 1 Canadia; 1 Canadia/New Zealand; 1 Fiji; 1 New Zealand/Hawaii
	3 Indigenous; 7 non-Indigenous

Semi-structured interviews were applied because as Adams [56] explains, this method is important where there is a gap in understanding of a topic. As this inquiry was about a new and evolving group tackling ecosystem approaches to health in Oceania, a gap existed in this research, requiring a method that could gather deeper understandings and allow for a robust discussion with participants [55]. Semi-structured interviews require the interviewer to: have knowledge of the topic; select information-rich and suitable participants; be empathetic and non-judgmental; listen carefully; use a checklist of questions; and pick an appropriate setting [56,57]. The semi-structured interview guide comprising 10 questions focused on:

1. Understanding the relationship between public health and environmental issues in Oceania;
2. The priorities, aspirations and challenges of the Oceania EcoHealth Chapter; and
3. How to incorporate Indigenous knowledge into the Oceania EcoHealth Chapter’s ethos.

Both a Plain Language Statement and Consent Forms were read, signed and agreed on by participants before data collection began. By agreeing to this, participants were interviewed face-to-face and audio recorded with data being transcribed and turned into a publication. All participants received a copy of their interview transcripts and could withdraw information collected. Interviews lasted approximately one hour.

Techniques such as mind mapping, axial and open coding, thematic and content analysis were employed to analyse the research data once several interviews had taken place [58,59,60]. Once the lead researcher had completed transcribing the interview, he read each of these documents a number of times to immerse himself in the data and draw out common themes by applying these approaches. Initial data analysis occurred in February to May (2014) with codes refined and tested from April, 2014 onwards. A co-author of this paper tested the reliability of this data every two months over a period of a year to ensure the themes and codes were reflective of the interviews.

## 5. Results

The results provide a snapshot of how Oceania EcoHealth Chapter members perceive the relationship between the environment and public health and the priorities, challenges and aspirations of this group.

### 5.1. Public Health and the Environment

All participants acknowledged the central role public health plays in tackling environmental issues. This is a reciprocal relationship “given that environmental issues will impact on public health” (Participant 7) and “sustainability and wellbeing are integrally linked” (Participant 2). Participants identified that these concepts cannot be separated being “wrapped up in the public health agenda” (Participant 6) and “co-dependent. It’s not possible… to address health of people, place, planet, without consideration of these intersections” (Participant 3). Participant 5 discussed this nexus highlighting:

“I’m really interested in the interface between the environment and social dynamics… sometimes I refer to that as socio-ecological systems, sometimes I stick with the notion of the environment embedded in broader ecosystems but I think at its core it’s a fundamental but neglected element of public health and that it’s been constrained with a fairly toxicological hazard focussed approach that doesn’t… capture the richness of those relationships”.

Participants viewed the role of public health practitioners in ecological sustainability is to “interact with the environmental actors, to inform them of the public health consequences of environmental change” (Participant 1). Participants highlighted that the public health and environment workforce need to act as advocates for ecosystem approaches to health. Participants frequently mentioned a method to influence this public health agenda was to show the link between nature and health. As Participant 9 mentioned:

“Using the green spaces that we have available, in cities, as places where you can reconnect with nature… Public health practitioners can think about how to do sustainability work in the context of built up areas… to promote close proximity to food to plate”.

However, Participant 8 said “many public health workers haven’t got a clue about the impact of… healthy landscapes”. Most participants noted public health is still conceived in silos, which “separates” and “divides” ideas of environment and health. As participant 5 noted:

“Public health is dominated by discourse around social determinants of health… I’m interested in the interface between the environment and social dynamics… it’s a… neglected element of public health… If you look at the Commission for Social Determinants of Health, the relationship with the environment… was mostly in relation to the built environment, there was little in relation to any of the relationships between the way in which environment and natural resources provide a foundation for socioeconomic factors… it made this big deal about climate change… [but] framing it in a distanced way… [At the] same time [the Millennium Ecosystem Assessment was released] and these two documents don’t even cross-reference each other”.

Participant 5 went on to say this epitomises the false dichotomy that exists between society, health and ecosystems, which does not emphasise the dynamic and holistic nature of this relationship. All participants noted this association is made clear and emphasised in Indigenous philosophies in Oceania. Participant 9 linked ecosystems and public health to Indigenous Australian peoples’ connection to traditional lands (known as Country), explaining:

“In Cape York… you have a different kind of connection to Country than… deserts because water’s plentiful… you know which part of the geography people come from because of height and stature… when you go through their Country with them, they just talk with such reverence... [with] a very close relationship between food, access, spirituality, Country… People map themselves into that Country differently. Out in Central Australia… people map themselves into their own Country through sunlight and where the shadows fall. In the Torres Strait, it’s navigational through stars… living on a small island communities, you were… aware of territories and boundaries because a multitude of people had to live together in close proximity with finite resources. So you couldn’t over-utilise them… when you start to think about that being the point of replication for societies across the whole of the world… how can billions of people all adopt a care for Country principle? When so many of us now are living in stressful situations… the impacts on wildlife and people’s lived experiences in different countries is so enormous. It’s going to be hard… to put that care for Country principle from within my own experience, into practice across the world, because we had a clear understanding… about how you live in Country. You can only live in what the ecosystem supports… At the moment, what we’re doing is living outside of the bounds of our ecosystems… That is going to be the major public health issue going forward”.

This section highlights the importance of the land, sea or water not only for Indigenous Australians but in reference to public health issues in Oceania.

### 5.2. Oceania EcoHealth Chapter Priorities

Participants saw the Chapter as a platform for incorporating diverse knowledge associated with ecosystems in Oceania. Participants highlighted the significant connections Indigenous populations have across Oceania and that the current ecological challenges facing the region will mirror what will happen across the globe in the future. Participant 6 highlighted:

“I think EcoHealth is very much a part of a local regional and global movement that seeks to redress the issues that we’re currently facing… recognising the instruction that Indigenous cultures can give us”.

Participant 1 urged the Chapter to incorporate Pacific Island perspectives because “a third of the Earth’s surface, and… economic concerns are governed by Pacific Islands”. This participant combined this knowledge through an “integrated holistic framework” but was frustrated that often groups like the Chapter were white-centric, requesting “more Indigenous perspectives and effort to merge the best of western science with traditional ecological knowledge”. Participants believed the Oceania EcoHealth Chapter was a platform for incorporating diverse “Aboriginal ways of seeing the world”. Therefore, Participant 3 noted that incorporation of this knowledge doesn’t just occur:

“through direct participation with Indigenous voices and the stories being captured… but also in exploring and engaging in what is indigeneity… as a collective, but also in a reflective personal spaces, and not taking the off the shelf options that are currently available… from a binary of Indigenous versus non-Indigenous… As an ongoing commitment to not being caught in the history of conflict, but learning from it”.

Participants mentioned the Chapter has the ability to cut across disciplines. Participant 2 noted that there are many disciplines “all talking about the same thing, and yet I've not heard this term EcoHealth… there’s a role there to draw together all those areas… to develop a common language”. All Participants believed the Chapter could be a platform to break disciplinary silos. Participant 6 explained:

“I had this remarkable experience with a bunch of Paleo-Ecologists… I presented a reconstruction of the past based on a sedimentary record, and a reconstruction of the past built on a Dreaming story [Indigenous Australian stories of the creation of the earth]… they both explain the creation of the landscaping…Then, a colleague of mine… said, “I would much prefer to believe an empirical record as a source of evidence,” and we talked through that… where we got to was the recognition, the value of a language that’s developed in place over… tens of thousands of years, and inbuilt in that language is a recognition of the place… we turned the worldview of his around by looking at language”.

Participants mentioned the priority of the Chapter was to provide “practical experience”, “sharing of knowledge”, and “opportunity for exchange” to bring “actions and activities together” and “explore beyond what we already know”. Participant 5 mentioned that the Chapter makes an “explicit effort to engage” in ecosystem approaches to health regionally having “the possibility to be strengthening… profiling and giving extra expression to an incredible body of work that’s been happening in this part of the world”. Perhaps this is because the group is “a loose association of affiliated people… I suspect, non-Indigenous people who join the Oceania Ecohealth Chapter—you’re on the fringes… capable of sharing through an equitable relationship rather than through a hierarchical way” (Participant 9). This space allows discussion like how we:

“Wrongly ascribe to environment—that it’s degraded... People who live closest to the environments are broken. But… Oceania has got a profound opportunity to really be influential in that space. How to promote the equality of diversity of lived experiences, how to maximise cultural flows of all people and communities who live there, but also then how to be respectful to all of the diversity of the species with whom we share on earth” (Participant 9).

Participant 7 highlighted regional issues will need to defined through “collective learning… but you have to start somewhere with Oceania there’s no question that the ocean and water” are central. All participants recognised that this needed to shift to strength-based approaches rather than deficit models to ignite the hearts and minds of people. Participants noted that the Chapter encourages learning and sharing. Participant 5 provided a reflection of embracing diverse Indigenous cultural knowledge:

“Obviously there is Indigenous context in most parts of the world but there is a particular array of those issues in the Oceania region… they sort of trip over the word EcoHealth… it’s a useful umbrella but I’m very comfortable with more context specific language emerging… there’s just this bubbling opportunity and the Oceania EcoHealth Chapter…”.

Participant 10 backed this statement up mentioning:

“EcoHealth can show how resolving Indigenous health issues can show us all a way forward... Finding a way to think collectively as us all embedded in a complex social-ecological system”.

These results reflect that EcoHealth approaches are a mechanism for incorporating diverse knowledge systems, traditional practices and experiences across Oceania.

### 5.3. Aspiration of the Oceania EcoHealth Chapter

“I’m hoping the Chapter will enable us… to move into spaces where there is no certainty, to be able to be vulnerable and comfortable with that” (Participant 3).

Participants mentioned various aspirations of the Chapter to move comfortably into uncertain spaces. Participant 1 wanted to “bridge the gap between environmental stewardship and public health”. Other participants saw the Chapter building a community with Participant 3 noting that it could bring us together to walk “more confidently forward… [to] challenge the constructions that we’ve all been educated in, in disciplinary silence without throwing the baby out with the bathwater, seeing what can emerge”. Participant 9 saw this occurring stating that the Chapter crosses “disciplines, cultures, continents, ages, and understandings of what it means to be in an environment in terms of a geographic perspective… hav[ing] respect for our Elders from Indigenous, organic and academic communities”.

Participants suggested that the Chapter offered a different way of viewing the world mentioning Indigenous values were critical. Participant 10 provided an example of this, noting “EcoHealth can show how resolving Indigenous health issues can show us all a way forward... Finding a way to think collectively as us all embedded in a complex social-ecological system… this will require some major mind-shifts”. Participant 2 saw the mechanism of the Oceania EcoHealth Chapter as acting “as interpreters between Aboriginal beliefs… values and culture as a translation service”. Further, participant 8 noted “there's still racial prejudice existing… EcoHealth could be a leader in pulling through and involving Indigenous people”. Other participants noted that until the Chapter has some “power”, these actions will not become a reality. Participant 5 saw this power shift, but did not agree the primary role of EcoHealth Chapter is to incorporate Indigenous values, through fostering:

“Different efforts that are connecting people back to Country… the chapter is going to struggle with the same tensions that any area of work does around notions of representation and engagement… that can be one of the main strategies to rectify… through place based engaged processes... that knowledge is richer because it’s happened in places of differences as well as similarities and we all learn from those contrasts… learning has intrinsic value irrespective”.

This highlights the importance of local based approaches that can guide global action. As Participant 6 noted that the aims and objectives of the Oceania EcoHealth Chapter align with the global EcoHealth movement but there is “something about recognising what we’re doing as a region more effectively… because it’s missing at the moment... we tend not to organise our thoughts around that regional level, so it’s redressing that imbalance”. A number of participants talked about the Chapter contributing to transformative change with Participant 9 noting:

“What we need is transformation… We’ve come together because we understand that things need to be different. We need to… draw on the core of our humanity to make that change. That’s why I think this Oceania EcoHealth Chapter will be a vehicle of radical hope”.

This section highlights that regionalism was perceived as providing a mechanism for community engagement.

## 6. Discussion

Participants understood the explicit and intersecting relationship between ecosystems and health and urged public health practitioners to shift from separating health, environment, social, and cultural determinants. These results are substantiated in literature acknowledging that ecosystems are critical to health and EcoHealth approaches could be effective [3,4,6,17,18,24], especially in Oceania to engage communities. A mechanism participants identified for promoting action in this field (which supports literature [61,62]) is through highlighting the relationship between contact with nature and health. Although, this has been demonstrated in urban settings, it may not be applicable across Oceania were many populations already have strong ties with local ecosystems [27,28,38,44] and, therefore, regional approaches and groups like the Oceania EcoHealth Chapter need to incorporate diverse social and ecological relationships into long-term approaches that engage communities [30,31,32].

The findings also validated calls for public health clinicians to become aware of the dynamics of determinants going beyond siloed approaches [7,13] and social determinants of health that dominate current discourse. Participants recognised that holistic Indigenous philosophies could be a mechanism for such change in Oceania. This offers a platform for the Oceania EcoHealth Chapter to advocate for ecosystem approaches to health, with participants identifying current strategies lacking understanding of the relationship between health and environment that embrace Indigenous knowledge (explored in a separate article by the authors [63]). Nonetheless, this mechanism allows for a deeper understanding of the relationship between sea, land, and water for diverse populations of Oceania.

The strength of the Oceania EcoHealth Chapter is its ability to cross disciplinary divides. The qualitative data highlights that members of Oceania EcoHealth Chapter aim to create a medium that Max-Neef [23] classifies as strong transdisciplinarity to allow for “radical change” and deeper understanding of realities required to tackle complex environmental and public health issues. For example, Participant’s 6 discussions with a paleo-ecologist to merge cultural understandings and language offered valuable insights on how to merge Indigenous and non-Indigenous knowledge. Participant 3 more broadly explained moving away from ‘disciplinary silence’ that Max-Neef [23] explain as working in “isolation” to practicing ecosystem approaches to health. Maclellan [33] acknowledged that alternative approaches are being focused on in Oceania because current mechanisms lack effectiveness. This supports literature [29,30,31,32,48] that recommends regional application be based on community involvement, long-term approaches, and the incorporation of a diversity of perspectives.

Participants mentioned the Chapter provided opportunity for discussions that could shift public consensus on environmental stewardship and public health. This further supports the EcoHealth value of transdisciplinary action. A key finding of this paper was the significance of cultural diversity in the Oceania region, with participants highlighting the importance of integrating multiple Indigenous knowledge systems which supports literature in the EcoHealth field [7,8,20]. However, participants recognised there should be a focus on representation and engagement with Indigenous peoples’ for full incorporation in the Oceania EcoHealth Chapter. The question for groups working in Oceania is whether they represent the region.

Participants recommended that the Chapter could provide tools to facilitate appropriate engagement with diverse communities. An example constantly provided by participants of applying a strength-based approach was the incorporation of Indigenous ecological knowledge as a positive platform for applying ecosystem approaches to health. Participants recognised a great deal of work is already occurring in Oceania so the Chapter’s role is not to create new systems but to bring people together to collectively engage, advocate, and provide diverse ways to understand health, ecosystems and social wellbeing.

Although diversity of understandings and knowledge of ecosystems were raised as priorities of the Chapter, there was acknowledgment that key themes in the region are oceans and water. Perhaps this is because of the historical and geographical relationship the Oceania region has with land, sea, and water systems [4,26,27,31,39,64].

Participants identified groups like the Oceania EcoHealth Chapter as a platform for merging environmental stewardship and public health by providing a framework for action that crosses disciplines, cultures and continents. Results from this research should be viewed only as a guideline due to the low number of participants and co-authors of this paper being Oceania EcoHealth Chapter members. Nonetheless, it is interesting that all participants in this research believed that by incorporating diverse regional perspectives, a positive transformation could occur to tackle global environmental and health issues. This shift supports the Lancet’s call for public health clinicians to become planetary health specialists [54]. The authors of this paper believe the Chapter could be one pathway to touch the minds and hearts of people towards ‘radical hope’ and planetary thinking by applying a regional focus to a global issue.

## 7. Conclusions

This paper indicated that there is a need for the spheres of public health and the environmental stewardship to be merged in practice but simultaneously ensuring that the diversity of perspectives throughout Oceania are considered. The priorities of the Oceania EcoHealth Chapter are to incorporate and engage local knowledge at a regional scale into the stewardship of land, water, and sea. The paper emphasises the importance of regional EcoHealth approaches that provide holistic and transdiciplinary mechanisms for local participation. Such approaches can provide tools to organisations working in Oceania to make a paradigm shift to community led initiatives that encourage an “eco-community”.

## References

[1] Arabena K. (2009). Policy Imagination: The Possibilities of Synthesis for EcoHealth Practitioners.

[2] Arebena K. (2015). Becoming Indigenous to the Universe.

[3] Chivian E. (2001). Environment and health: 7. Species loss and ecosystem disruption—the implications for human health. Can. Med. Assn. J..

[4] Horwitz P., Finlayson C.M. (2011). Wetlands as settings for human health: Incorperating ecosystem services and health impact assessment into water resource management. Bio. Sci..

[5] Frumkin H. (2002). Urban sprawl and public health. Publ. Health Report.

[6] Clark N.E., Lovell R., Wheeler B.W., Higgins S.L., Depledge M.H., Norris K. (2014). Biodiversity, cultural pathways, and human health: A framework. Trend Ecol. Evolut..

[7] Parkes M. (2010). EcoHealth and Aboriginal health: A review of common ground.

[8] Arabena K., Kingsley J., Walker R. (2015). Climate change: Impact on country and aboriginal and Torres Strait Islander culture. Climate Change Adaptation for Health and Social Services.

[9] (2012). Resolution XI.12: Wetlands and Health: Taking an Ecosystem Approach. Proceedings of 11th Meeting of the Conference of the Parties to the Convention on Wetlands.

[10] Pawu-Kurlpurlurnu W.J., Holmes M., Box A.L. (2008). Ngurra-Kurlu: A way of working with Warlpiri people.

[11] Holmes M., Jampijinpa W. (2013). Law for country: The structure of Warlpiri ecological knowledge and its application to natural resource management and ecosystem stewardship. Eco. Soc..

[12] International Association for Ecology and Health. http://www.ecohealth.net/association.php.

[13] Webb J.C., Mergler D., Parkes M.W., Saint-Charles J., Spiegel J., Waltner-Toews D., Yassi A., Woollard R.F. (2010). Tools for thoughtful action: The role of ecosystem approaches to health and enhancing public health. Can. J. of Public Health.

[14] Bronfenbrenner U. (1979). The Ecology of Human Development: Experiments by Nature and Design.

[15] Hancock T., Perkins F. (1985). The mandala of health: A conceptual model and teaching tool. Health Educ..

[16] World Health Organisation (1986). Ottawa Charter for Health Promotion.

[17] Albrecht G., Higginbotham N., Connor L., Freeman S., Heggenhougen K., Quah S. (2008). Social and cultural perspectives on Eco-Health. International Encyclopaedia of Public Health.

[18] Wilcox B., Kueffer C. (2008). Transdisciplinarity in EcoHealth: Status and future prospects. EcoHealth.

[19] Charron D.F., Charron D.F. (2012). EcoHealth: Orgins and Approaches. Ecohealth Research in Practice: Innovative Applications of an Ecosystem Approach to Health.

[20] Saint-Charles J., Webb J., Sanchez A., Mallee H., de Joode B.W., Nguyen-Viet H. (2014). EcoHealth as a field: Looking forward. EcoHealth.

[21] Parkes M.W., Charron D.F., Sanchez A., Charron D.F. (2012). Better Together: Field-Building Networks at the Frontiers of EcoHealth Research. EcoHealth Research in Practice: Innovation Applications of an Ecosystem Approach to Health, Insight and Innovation in International Development.

[22] Parkes M. (2012). Diversity, emergence, resilience: Guides for a new generation of EcoHealth research and practice. EcoHealth.

[23] Max-Neef M.A. (2005). Foundations of transdisciplinarity. Ecol. Econ..

[24] Lovell R., Wheeler B.W., Higgins S.L., Irvine K.N., Depledge M.H. (2014). A systemic review of the health and well-being benefits of biodiverse environments. J. Toxicol. Environ. Health.

[25] Kingsley J., Lawson J. (2015). Finding a unified understanding of nature. EcoHealth.

[26] Wardell-Johnson G.W., Keppel G., Sander J. (2011). Climate change impacts on the terrestrial biodiversity and carbon stocks of Oceania. Pacific Conserv. Bio..

[27] Jupiter S., Mangubhai S., Kingsford R.T. (2014). Conservation of biodiversity in the Pacific islands of Oceania: Challenges and opportunities. Pacific Conserv. Bio..

[28] Grantham H.S., McLeod E., Jupiter S.D., Hardcastle J., Richardson A.J., Poloczanska E.S., Hills T., Mieszkowska N., Klein C.J., Watson J.E.M. (2011). Ecosystem-based adaptation in marine ecosystems of tropical Oceania in response to climate change. Pacific Conserv. Bio..

[29] Hudson R. (2005). Region and place: Devolved regional government and regional economic success?. Prog. Hum. Geogr..

[30] Lewis S., Kouri D. (2004). Regionalization: Making sense of the Canadian experience. Healthcare.

[31] Burns W.C.G., Gleik P.H., Burns W.C.G., Chalecki E.L., Cohen M. (2002). Pacific Island developing country water resources and climate change. World’s Water 2002–2003: The Biennial Report of Freshwater Resources.

[32] Summers D.M., Bryan B.A., Meyer W.S., Lyle G., Wells S., McLean J., Moon T., van Gaans G., Siebentritt M. (2015). Simple models for managing complex social-ecological systems: The Landscape Future Analysis Tool (LFAT). Environ. Modell. Softw..

[33] Maclellan N. (2015). Transforming the regional architecture: New players and challenges for the Pacific Islands. Asia Pac..

[34] Rodriguez J., Vos F., Below R., Guha-Sapir D. (2009). Annual disaster statistical review 2008: The numbers and trends.

[35] Australian Bureau of Meteorology & CSIRO (2011). Climate Change in the Pacific: Scientific Assessment and New Research.

[36] Jenkins A.P., Jupiter S.D., Finlayson C.M., Horwitz P., Weinstein P. Natural disasters, health and wetlands: A Pacific small island developing state perspective. Wetlands and Human Health.

[37] (1997). Small Island States. Intergovernmental Panel on Climate Change. Regional impacts of climate change: An assessment of vulnerability.

[38] Woinarski J.C.Z. (2010). Biodiversity conservation in tropical forest landscapes of Oceania. Bio. Conserv..

[39] McMichael A.J., Woodruff R., Whetton P.H., Hennessy K., Nicholls N., Hales S., Woordward A., Kjellstrom T. (2003). Human health and climate change in Oceania: A risk assessment 2002.

[40] Nurse L.A., Sem G., Hay J.E., Suarez A.G., Wong P.P., Briguglio L., Ragoonaden S. (2001). Small Island States. Impact, adaptation and vulnerability.

[41] Pulver L.J., Haswell M.R., Ring I., Waldon J., Clark W., Whetung V., Kinnon D., Graham C., Chino M., LaValley J., Sadana R. (2010). Indigenous Health—Australia, Canada, Aotearoa New Zealand and the United States—Laying Claim to a Future that Embraces Health for us all.

[42] Kayser M. (2010). The human genetic history of Oceania: Near and remote views of dispersal. Curr. Biol..

[43] Hanna E.G., McIver L., Butler C.D. (2014). Small Island States—Canaries in the coal mine of climate change and health. Climate Change and Global Health.

[44] Duffy D.C. (2011). No room in the ark? Climate change and biodiversity in the pacific islands of Oceania. Pacific Conserv. Bio..

[45] Jenkins K.M., Kingsford R.T., Closs G.P., Wolfenden B.J., Matthaei C.D., Hay S.E. (2011). Climate change and freshwater ecosystems in Oceania: An assessment of vulnerability and adaptation opportunities. Pacific Conserv. Bio..

[46] Ahlgren I., Yamada S., Wong A. (2014). Rising Oceans, climate change, food aid, and human rights in the Marshall Islands. Health Hum. Rights.

[47] Kingsford R.T., Watson J.E.M. (2011). What hope for biodiversity in the face of anthropogenic climate change in Oceania?. Pacific Conserv. Bio..

[48] Jupiter S., Jenkins A., Lee Long W., Maxwell S., Carruthers T., Hodge K.B., Govan H., Tamelander J., Watson J. (2014). Principles for integrated island management in the tropical Pacific. Pacific Conserv. Bio..

[49] Nutbeam D. (1998). Health promotion glossary. Health Promot. International.

[50] Knight J., Germov J. (1999). Models of health. Second Opinion: An Introduction to Health Sociology.

[51] Jolley D., Keleher H., Murphy B. (2004). Major achievements in public health since 1850. Understanding Health: A Determinants Approach.

[52] Horton R., Beaglehole R., Bonita R., Raeburn J., McKee M., Wall S. (2014). From public to planetary health: A manifesto. Lancet.

[53] Bryman A. (2004). Social research methods.

[54] Lambert V., Glacken M., McCarron M. (2011). Employing an ethnographic approach: Key characteristics. Nurse Res..

[55] Minichiello V., Sullivan G., Greenwood K., Axford R. (2004). Handbook of Research Methods in Nursing and Health Science.

[56] Adams E. (2010). The joy and challenges of semi-structured interviewing. Com. Pract..

[57] Whiting L.S. (2008). Semi-structured interviews: Guidance for novice researchers. Nursing Stand..

[58] Creswell J.W. (1998). Qualitative Inquiry and Research Design: Choosing among Five Traditions.

[59] Browne J., Minichiello V., Sullivan G., Greenwood K., Axford R. (2004). Grounded theory analysis: Coming to data with questioning minds. Handbook of Research Methods for Nursing and Health Science.

[60] Harris J.E., Gleason P.M., Sheean P.M., Boushey C., Beto J.A., Bruemmer B. (2009). An introduction to qualitative research for food and nutrition professionals. J. Amer. Diet. Assn..

[61] Kingsley J., Townsend M. (2006). “Dig in” to social capital: community gardens as mechanisms for growing urban social connectedness. Urban Pollut. Res..

[62] Kingsley J., Townsend M., Henderson-Wilson C. (2009). Cultivating health and wellbeing: Members’ perceptions of the health benefits of a Port Melbourne community garden. J. Leisure Stud..

[63] Kingsley J., Patrick R., Parkes M., Arabena K., Horwitz P., Massey C., Jenkins A. (2015). Shifting public health: Incorporating diversity, ecosystems and Indigenous knowledge. EcoHealth.

[64] Strang V. (2009). Gardening the world: Agency, identity, and the ownership of water.

